# Angle-Only Cooperative Orbit Determination Considering Attitude Uncertainty

**DOI:** 10.3390/s23020718

**Published:** 2023-01-08

**Authors:** Yishuai Shi, Junkui Wang, Chuankai Liu, Yangjun Wang, Qingchao Xu, Xingyu Zhou

**Affiliations:** 1 School of Aerospace Engineering, Beijing Institute of Technology, Beijing 100081, China; 2 Department of Mathematics and Theories, Peng Cheng Laboratory, Shenzhen 518000, China; 3 Beijing Aerospace Control Center, Beijing 100094, China; 4 Key Laboratory of Sicence and Technology on Space Flight Dynamics, Beijing 100094, China

**Keywords:** cooperative orbit determination, angle-only measurement, observability matrix, constellation, cubature Kalman filter

## Abstract

In this paper, a novel concept for cooperative orbit determination (OD) using inter-spacecraft angle-only measurements is proposed. Different from the conventional cooperative OD that only estimates orbit states, the attitude of the observer spacecraft is considered by incorporating the attitude into the estimated vector. The observability of a two-spacecraft system is analyzed based on the observability matrix. Observability analysis reveals that inter-spacecraft angle-only measurements are inadequate to estimate both the attitude and the orbit states in two-body dynamics. The observability of the two-spacecraft system can be improved by considering high-order gravitational perturbation or executing an attitude maneuver on the observer spacecraft. This is the first time that we present the observability analysis and orbit estimation results for a two-spacecraft system considering attitude uncertainty for the observer. Finally, simulation results demonstrate the effectiveness of the proposed method. The results in this paper can be potentially useful for autonomous managements of a spacecraft constellation and formation.

## 1. Introduction

Autonomous orbit determination (OD), due to its considerable value in space systems engineering, has been of widespread interest in the last several decades [1,2,3,4]. The ability of spacecraft to determine their own states, without the help of ground-based tracking equipment, can improve their ‘intelligence’ and survivability and may also reduce operational management costs [5,6,7,8]. With the current plans and development of future multispacecraft constellations, the latter issue, i.e., efficient constellation management, becomes particularly important, and autonomous OD for a spacecraft constellation is highly desirable [9,10,11].

Filter algorithms have been widely used in many OD applications since R.E. Kalman proposed his famous recursive method Kalman filter (KF) to solve discrete linear filtering problems in 1960 [12,13,14]. Those filter algorithms could utilize the provided measurements coming from different sensors to obtain the required estimates of the orbit states [15,16,17,18]. Thus, current filter algorithms rely on the accurate measurement information measured by different sensors equipped on the spacecraft. In this situation, many filter-based methods have been proposed to solve the autonomous OD problem for a single spacecraft using GPS, radar, guidepost and magnetic field vector measurements [19,20,21,22]. Although the above methods have been proven to be efficient in providing considerable estimation, these measurements rely on complicated measurement equipment, which is impossible for constellation spacecraft.

Recently, a variety of methods have been proposed to solve the autonomous OD problem for a satellite constellation by using only interspacecraft relative measurements [23,24,25,26]. However, they all face the rank deficiency, meaning that the multispacecraft system with interspacecraft measurements alone is unobservable [27]. Interspacecraft ranging systems are widely used to obtain range-only measurements between two spacecraft, but they lack observability in some specific situations [23]. It has been concluded that in the multibody system, due to the nonsymmetric gravity from the noncentral gravitational body, all the orbit elements of two spacecraft can be obtained using only intersatellite range measurements [28,29]. However, intersatellite range measurements are not adequate to obtain all orbit elements in two-body dynamics because the rotation of the orbits with respect to the inertial reference system cannot be determined, and only the shape, size, and relative orientation of two orbits can be obtained using only intersatellite range measurements.

Another well-known method employs onboard cameras to obtain the relative angle measurements, which only include the relative direction information [24,25]. The angle-only navigation is simple, robust, and well proven in many applications [30]. However, the inertial states of the spacecraft cannot be observed without additional measurements. At least one beacon (usually noted as attitude-reference spacecraft) must be added to the system to ensure that there is a reference in inertial space or that other sources of measurements should be added [31]. The basic requirement for orbit determination using angle-only measurements is that the line-of-sight vector must be measured with respect to the inertial frame, which requires several types of sensors, such as a laser sensor to measure the relative range, an optical camera to measure the relative direction, and a star sensor to measure the inertial attitude [24,25,32]. However, due to the size, mass, and power consumption problem of active sensors such as star sensors, they might be unable to be on-boarded for small and microspacecraft to estimate the inertial attitude. Therefore, the inertial attitude of observing spacecraft should also be added into the OD problem when using intersatellite line-of-sight angle measurements. Fortunately, the conclusion of OD using angle-only measurements in two-body dynamics does not hold in other dynamical cases. Specifically, the obtainable orbit elements deviate from the prior conclusion under a more complex dynamic. For example, perturbations affect the orbits and contribute to deviations of real orbits from ideal two-body dynamics, which may improve the observability of a system. Moreover, with a calibrated thrust maneuver, observability can be guaranteed for possible situations [33].

Observability analysis is necessary to determine whether a particular measurement system is sufficient to solve the state estimation problem [34,35,36], and a number of researchers have carried out this analysis in the context of the OD problem [37,38,39]. One unwieldy approach to establish observability is to use classical nonlinear techniques that rely on Lie-derivative criterion methods [24,25]. In general, it is difficult to provide further results on the system observability based on the Lie derivative criterion because the analytical expressions of higher-order Lie derivatives become progressively more complicated. Hence, discussing observability for nonlinear systems using Lie algebra and differential geometry methods becomes quite difficult. Another type of approach for establishing observability is the observability matrix (OM) [1,11]. The OM method can be used to determine whether a system can be observed using sequential measurements, which are easy to obtain through numerical propagation.

In this paper, a novel concept for autonomous OD is proposed based on a two-spacecraft system with angle-only measurements. Differently from the traditional autonomous OD using intersatellite angle measurements, the inertial attitude of the observer is also added to be estimated. In this manuscript, we present for the first time the observability analysis and the orbit estimation results for a two-spacecraft system with different additional conditions. Five conditions are considered and for each condition, the observability of the OD system is investigated using the OM.

The remainder of this paper is organized as follows. The state model and the measurement model used is firstly presented in Section 2, followed by brief reviews of the OM and the fifth-order CKF. Five conditions, as well as the corresponding OMs, are discussed in Section 3. The observability analysis and filter estimation results are shown in Section 4. Finally, conclusions are given in Section 5.

## 2. Attitude and Orbit Determination Model

In this section, the basic mathematical model, namely, the state model and the observation model, for the autonomous attitude and orbit determination problem of a two-spacecraft system using angle-only measurements is first presented. In addition, the observability matrix is introduced to analyze the observability of the system. Finally, the widely used fifth-order cubature Kalman filter is reviewed for later estimation.

### 2.1. State Model

**Figure 1:**
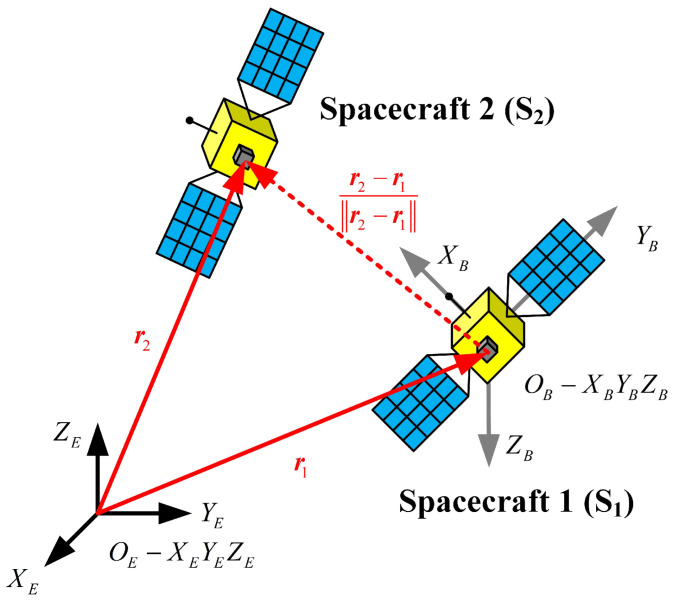
Diagram of a two-spacecraft system.

Two spacecraft, defined as $S_{1}$ and $S_{2}$, are considered and shown in Figure 1. Assume that $S_{1}$ is able to observe the line-of-sight angle between $S_{1}$ and $S_{2}$. Note that $S_{1}$ is not considered as the attitude-reference spacecraft, which is different from recent works.

The attitude-orbit determination developed in this paper aims to determine the absolute states (i.e., the absolute orbit of both $S_{1}$ and $S_{2}$, together with the absolute attitude of $S_{2}$) of the spacecraft system, which for Earth orbiting bodies are usually described in the Earth-centered inertial (ECI) frame (coordinate system $O_{e}-X_{e}Y_{e}Z_{e}$ in Figure 1). The analysis developed in this paper builds primarily on the two-body dynamics of the spacecraft with Earth as the primary body. Define the state of the *i*-th spacecraft in the ECI frame as:

(1) $$X_{i}={\left[r_{i}^{T},v_{i}^{T}\right]}^{T}={\left[x_{i},y_{i},z_{i},{\dot{x}}_{i},{\dot{y}}_{i},{\dot{z}}_{i}\right]}^{T}$$

The state equation of the spacecraft orbit under the two-body dynamics can be expressed in general form as:

(2) $${\dot{X}}_{i}=f\left(X_{i}\right)=\left[\begin{array}{c} \;{\dot{r}}_{i}\\ \;{\ddot{r}}_{i} \end{array}\right]$$

In the case of ideal two-body dynamics (i.e., particle dynamics model), Equation (2) is given by:

(3) $${\ddot{r}}_{i}=-\frac{\mu_{e}}{∥r_{i}∥}r_{i}$$

In the case of considering $J_{2}$, $J_{3}$, and $J_{4}$ perturbations, Equation (3) is rewritten as:

(4) $${\ddot{r}}_{i}=-\frac{\mu_{e}}{∥r_{i}∥}r_{i}+a_{J_{2}}+a_{J_{3}}+a_{J_{4}}$$

where $\mu_{e}$ is the Earth’s gravitational parameter, and $a_{J_{2}}$, $a_{J_{3}}$ and $a_{J_{4}}$ are the perturbation acceleration of $J_{2}$, $J_{3}$, and $J_{4}$, which can be obtained by derivative of potential function with respect to position. The potential function is given by:

(5) $$U=\frac{\mu }{r}\left[1-J_{2}{\left(\frac{R_{e}}{r}\right)}^{2}P_{2}\left(\sin\varphi \right)-J_{3}{\left(\frac{R_{e}}{r}\right)}^{3}P_{3}\left(\sin\varphi \right)-J_{4}{\left(\frac{R_{e}}{r}\right)}^{4}P_{4}\left(\sin\varphi \right)\right]$$

where φ is the latitude of the spacecraft’s ground trace; $P_{2}\left(\sin\varphi \right)=\frac{3}{2}{\left(\sin\varphi \right)}^{2}-\frac{1}{2}$, $P_{3}\left(\sin\varphi \right)=\frac{5}{2}{\left(\sin\varphi \right)}^{3}-\frac{1}{2}\left(\sin\varphi \right)$ and $P_{4}\left(\sin\varphi \right)=\frac{35}{8}{\left(\sin\varphi \right)}^{4}-\frac{30}{8}{\left(\sin\varphi \right)}^{2}+\frac{3}{8}$.

The attitude of spacecraft $S_{1}$ is represented through the quaternion, defined by:

(6) $$q=\left[\begin{array}{c} q_{0}\\ q_{13} \end{array}\right]$$

where:

(7) $$q_{13}=\left[\begin{array}{c} q_{1}\\ q_{2}\\ q_{3} \end{array}\right]=\hat{n}\sin\left(\alpha /2\right)=\hat{n}\sin\left(\alpha /2\right)$$

(8) $$q_{0}=\cos\left(\alpha /2\right)$$

where $\hat{n}$ is a unit vector corresponding to the principal axis of rotation and α is the angle of rotation. The quaternion kinematics are derived through the spacecraft’s angular velocity as follows:

(9) $$\dot{q}=g_{1}\left(q\right)=\frac{1}{2}\Omega \left(\omega \right)q$$

(10) $$\dot{\omega }=g_{2}\left(\omega \right)=J^{-1}\left(\omega^{\times }J\omega +M\right)$$

where $\omega ={\left[\omega_{x}\;\omega_{y}\;\omega_{z}\right]}^{T}$ denotes the angular velocity, J represents the moment of inertia of the spacecraft, M is the sum of external moments, and matrix Ω(ω) is defined as:

(11) $$\Omega \left(\omega \right)=\left[\begin{array}{cc} 0 & -\omega^{T}\\ \omega & -\omega^{\times } \end{array}\right],\omega^{\times }=\left[\begin{array}{ccc} 0 & -\omega_{z} & \omega_{y}\\ \omega_{z} & 0 & -\omega_{x}\\ -\omega_{y} & \omega_{x} & 0 \end{array}\right]$$

The four quaternion elements satisfy the following normalization constraint:

(12) $$q^{T}q=q_{13}^{T}q_{13}+q_{0}^{2}=1$$

Combining the elements for the above orbits and attitude, the state vector to be estimated is:

(13) $$\begin{array}{c} X={\left[X_{1}^{T},X_{2}^{T},q^{T},\omega^{T}\right]}^{T}\\ ={\left[x_{1},y_{1},z_{1},{\dot{x}}_{1},{\dot{y}}_{1},{\dot{z}}_{1},x_{2},y_{2},z_{2},{\dot{x}}_{2},{\dot{y}}_{2},{\dot{z}}_{2},q_{0},q_{1},q_{2},q_{3},\omega_{x},\omega_{y},\omega_{x}\right]}^{T} \end{array}$$

The state model is given by:

(14) $$X=F\left(X\right)={\left[{\dot{X}}_{1}^{T},{\dot{X_{2}}}^{T},{\dot{q}}^{T},{\dot{\omega }}^{T}\right]}^{T}={\left[f{\left(X_{1}\right)}^{T},f{\left(X_{2}\right)}^{T},g_{1}{\left(\dot{q}\right)}^{T},g_{2}\left(\omega \right){}^{T}\right]}^{T}$$

### 2.2. Observation Model

As shown in Figure 1, the spacecraft $S_{2}$ is assumed to be observed by the spacecraft $S_{1}$. The inertial inter-spacecraft angle measurements can be obtained if the observer $S_{1}$ is an attitude-reference spacecraft. The inertial inter-spacecraft angle measurements can be represented by two angulars α and β, given as:

(15) $$\left\{\begin{array}{c} \alpha =\tan^{-1}\left(\Delta y/\Delta x\right)+\varepsilon_{\alpha }\\ \beta =\sin^{-1}\left(\Delta z/\sqrt{\Delta x^{2}+\Delta y^{2}+\Delta z^{2}}\right)+\varepsilon_{\beta } \end{array}\right.$$

where $\Delta x=x_{2}-x_{1}$, $\Delta y=y_{2}-y_{1}$ and $\Delta z=z_{2}-z_{1}$; $\varepsilon_{\alpha }$ and $\varepsilon_{\beta }$, respectively, denote the randomly distributed noise for the two angulars. To be convenient, the angular measurement equations in Equation (15) can be further replaced by the line-of-sight model, given by:

(16) $$y^{\prime }=\frac{r_{2}-r_{1}}{∥r_{2}-r∥}+\varepsilon^{\prime }$$

where $r_{1}={\left[x_{1},y_{1},z_{1}\right]}^{T}$ and $r_{2}={\left[x_{2},y_{2},z_{2}\right]}^{T}$; $\varepsilon^{\prime }$ denotes the randomly distributed noise for vector measurement $y^{\prime }$.

Note that in this paper, the attitude of spacecraft $S_{1}$ also needs to be estimated. Therefore, $S_{1}$ can only measure the line-of-sight angle in the spacecraft body coordinate system (i.e., coordinate system $O_{B}-X_{B}Y_{B}Z_{B}$), and the real measurement y is given by the following:

(17) $$y=R_{E}^{B}y^{\prime }+\varepsilon =R_{E}^{B}\frac{r_{2}-r_{1}}{∥r_{2}-r_{1}∥}+\varepsilon$$

where ε is the corresponding measurement noise vector characterized by a normal distribution with zero mean and covariance $R\in R^{3\times 3}$ and $R_{E}^{B}$ is the rotation matrix from ECI (coordinate system $O_{E}-X_{E}Y_{E}Z_{E}$) to the spacecraft body coordinate system $O_{B}-X_{B}Y_{B}Z_{B}$:

(18) $$R_{E}^{B}=\left[\begin{array}{ccc} q_{0}^{2}+q_{1}^{2}-q_{2}^{2}-q_{3}^{2} & 2\left(q_{0}q_{3}+q_{1}q_{2}\right) & 2\left(q_{1}q_{3}-q_{0}q_{2}\right)\\ 2\left(-q_{0}q_{3}+q_{1}q_{2}\right) & q_{1}^{2}-q_{1}^{2}+q_{2}^{2}-q_{3}^{2} & 2\left(q_{2}q_{3}+q_{q_{1}}q_{1}\right)\\ 2\left(q_{1}q_{2}+q_{1}q_{3}\right) & 2\left(q_{2}q_{3}-q_{0}q_{1}\right) & q_{0}^{2}-q_{1}^{2}-q_{2}^{2}+q_{3}^{2} \end{array}\right]$$

Hence, the navigation problem is given by:

(19) $$\left\{\begin{array}{c} \dot{X}={\left[{\dot{X}}_{1}^{T},{\dot{X}}_{2}^{T},{\dot{q}}^{T}\right]}^{T}={\left[f{\left(X_{1}\right)}^{T},f{\left(X_{1}\right)}^{T},g{\left(q\right)}^{T}\right]}^{T}\\ y=R_{E}^{B}\frac{r_{2}-r_{1}}{∥r_{2}-r_{1}∥}+\varepsilon \end{array}\right.$$

### 2.3. Observability Matrix

In this paper, the observability matrix (OM) is taken as a metric to evaluate the feasibility of the two-spacecraft system. With measurements collected *k* times sequentially, denoted as $\left\{y_{0},y_{1},\cdots ,y_{k-1}\right\}$ from time epoch $t_{0}$ to $t_{k-1}$, the OM is represented as:

(20) $$N=\left[\begin{array}{c} {\tilde{H}}_{0}\\ \vdots \\ {\tilde{H}}_{k-1} \end{array}\right]$$

(21) $${\tilde{H}}_{k}=H_{k}\Phi \left(t_{k},t_{0}\right)$$

where $\;\;\;\Phi \;\;\;(t_{k},t_{0})$ is the state transformation matrix (STM) from $t_{0}$ to $t_{k}$ and

(22) $$H_{k}={\left.\frac{\partial y}{\partial X}\right|}_{t=t_{k}}$$

The differential equation of the STM is as follows:

(23) $$\dot{\Phi }\left(t_{k},t_{0}\right)={\left.\frac{\partial F(X)}{\partial X}\right|}_{t=t_{k}}\Phi \left(t_{k},t_{0}\right)$$

and is initialized by:

(24) $$\Phi \left(t_{0},t_{0}\right)=I_{n_{\times }n}$$

where *n* is the dimension of the state vector to be estimated and $I_{n\times n}$ is an *n*-dimensional unit matrix. Note that the differential term ∂F(X)/∂X is also known as the Jacobi matrix of state model (14).

An OM with a full rank (i.e., rank(N)=n) indicating that the two-spacecraft system is observable using the given measurements [1]. Moreover, the observability degree of the two-spacecraft system can be described by the condition number (CN) of OM, represented by $\mathrm{cond}\left(N\right)=∥N∥\cdot ∥N^{-1}∥$. The smaller the CN, the better the observability [24].

### 2.4. Review of Fifth-Order CKF

In this section, the fifth-order CKF algorithm is briefly summarized. First, consider a discrete nonlinear system as:

(25) $$\begin{array}{c} x_{k}=f\left(x_{k-1}\right)+w_{k-1}\\ z_{k}=h\left(x_{k}\right)+v_{k} \end{array}$$

where $x_{k}\in R^{n}$ is the state vector at time epoch *k* and $z_{k}\in R^{m}$ is the measurement. $w_{k-1}\in R^{n}$ and $v_{k}\in R^{m}$ denote the independent system and measurement noise, respectively, and are both considered independent and white Gaussian distributions with covariances $Q_{k-1}$ and $R_{k}$, respectively.

The optimal Bayesian filters contains two steps: the prediction step and the update step. Both of the two steps require us to calculate the Gaussian weighted integration $\int_{R^{n}}g\left(x\right)N\left(x;\bar{x},P\right)dx$, where g(x) is a nonlinear function. The integral with respect to the general Gaussian distribution $N(x;\bar{x},P)$ can be further approximated by:

(26) $$\begin{array}{cc} \int_{R^{n}}g\left(x\right)N\left(x;\bar{x},P\right)dx & =\int_{R^{n}}g\left(Ax+\bar{x}\right)N\left(x;\bar{x},P\right)dx\\ \; & \approx \sum_{i=1}^{N_{p}}W_{i}g\left(A\gamma_{i}+\bar{x}\right) \end{array}$$

where $P=SS^{T}$, $N_{P}$ is the total number of points, and $\gamma_{i}$ and $W_{i}$ are the quadrature points and weights, respectively, corresponding to the Gaussian distribution $N(x;\bar{x},P)$. Specifically, the integral with respect to N(x;0,I) can be approximated by the following quadrature rule:

(27) $$\int_{R^{n}}g\left(x\right)N\left(x;0,I\right)dx\approx \sum_{i=1}^{N_{p}}W_{i}g\left(\gamma_{i}\right)$$

According to the cubature rule and Equation (26), in fifth-order CKF, the cubature points $\zeta_{i}$ are given by [21]:

(28) $$\left\{\begin{array}{c} \zeta_{0}=0_{n\times 1}\\ \zeta_{i}=A_{k}\cdot \sqrt{n+2}e_{i}\\ \zeta_{i+n}=-A_{k}\cdot \sqrt{n+2}e_{i}\\ \zeta_{j+2n}=A_{k}\cdot \sqrt{n+2}s_{j}^{+}\\ \zeta_{j+2n+n\left(n-1\right)/2\;}=-A_{k}\cdot \sqrt{n+2}s_{j}^{+}\\ \zeta_{j+2n+n\left(n-1\right)}=A_{k}\cdot \sqrt{n+2}s_{j}^{-}\\ \zeta_{j+2n+3n\left(n-1\right)/2\;}=-A_{k}\cdot \sqrt{n+2}s_{j}^{-}\\ i=1,2,\ldots ,n\;\;;\;\;j=1,2,\ldots ,n\left(n-1\right)/2\; \end{array}\right.$$

where *n* is the state dimension of the system to be estimated, $A_{k}$ is the Cholesky decomposition of covariance matrix $P_{k}$ at epoch $t_{k}$ and $P_{k}=A_{k}A_{k}^{T}$, and $e_{i}$ is the *i*-th column of the *n*-th identity matrix $I_{n}$. The point sets $s_{j}^{+}$ and $s_{j}^{-}$ are given as follows.

(29) $$\begin{array}{cc} \; & \left\{s_{j}^{+}\right\}=\left\{\left(e_{p}+e_{q}\right)/\sqrt{2}\;\left|p<q,p,q=1,2,\ldots ,n\right.\right\}\\ \; & \left\{s_{j}^{-}\right\}=\left\{\left(e_{p}-e_{q}\right)/\sqrt{2}\;\left|p<q,p,q=1,2,\ldots ,n\right.\right\} \end{array}$$

The corresponding weight $w_{i}$ of each Cubature point $\zeta_{i}$ is given by

(30) $$w_{i}=\left\{\begin{array}{cc} \frac{2}{n+2} & i=0\\ \frac{1}{{\left(n+2\right)}^{2}} & i=1,\ldots ,2n(n-1)\\ \frac{4-n}{{\left(n+2\right)}^{2}} & i=2n\left(n-1\right)+1,\ldots ,2n^{2} \end{array}\right.$$

Then, the fifth-order CKF algorithm is summarized as follows:

i. Time updating:

(31) $$\begin{array}{cc} \; & A_{k}=Cholesky\left(P_{k-1|k-1}\right)\\ \; & \chi_{i,k-1|k-1}=\zeta_{i}+x_{k-1|k-1}\\ \; & \chi_{i,k|k-1}^{*}=f(\chi_{i,k-1|k-1})\\ \; & x_{k|k-1}=\sum_{i=0}^{2n^{2}}w_{i}\chi_{i,k|k-1}^{*}\\ \; & P_{k|k-1}=\sum_{i=0}^{2n^{2}}w_{i}\left(\chi_{i,k|k-1}^{*}-x_{k|k-1}\right){\left(\chi_{i,k|k-1}^{*}-x_{k|k-1}\right)}^{T}+Q_{k} \end{array}$$

ii. Measurement updating

(32) $$\begin{array}{cc} \; & A_{k}=Cholesky\left(P_{k|k-1}\right)\\ \; & \chi_{i,k|k-1}=\zeta_{i}+x_{k|k-1}\\ \; & z_{i,k|k-1}^{*}=h(\chi_{i,k|k-1})\\ \; & z_{k|k-1}=\sum_{i=0}^{2n^{2}}w_{i}z_{i,k|k-1}^{*}\\ \; & P_{\mathrm{xz},k|k-1}=\sum_{i=0}^{2n^{2}}w_{i}\left(\chi_{i,k|k-1}^{*}-x_{k|k-1}\right){\left(z_{i,k|k-1}^{*}-z_{k|k-1}\right)}^{T}\\ \; & P_{\mathrm{zz},k|k-1}=\sum_{i=0}^{2n^{2}}w_{i}\left(z_{i,k|k-1}^{*}-z_{k|k-1}\right){\left(z_{i,k|k-1}^{*}-z_{k|k-1}\right)}^{T}+R_{k}\\ \; & K_{k}=P_{\mathrm{xz},k|k-1}P_{\mathrm{zz},k|k-1}^{-1}\\ \; & x_{k|k}=x_{k|k-1}+K_{k}(z_{k}-z_{k|k-1})\\ \; & P_{k|k}=P_{k|k-1}-K_{k}P_{\mathrm{zz},k|k-1}K_{k}^{T} \end{array}$$

where *Cholesky*(·) represents the Cholesky decomposition method, $\chi_{i,k|k-1}^{*}$ is the Cubature point generated from states and $z_{i,k|k-1}^{*}$ represents the Cubature point generated from measurements.

## 3. Attitude and Orbit Determination Method with Angle-Only Measurements

In this section, the autonomous attitude and orbit determination problem, with different conditions, are modeled based on the theory in Section 2.

### 3.1. Case I: Both Orbits of $S_{1}$ and $S_{2}$ Are Known, and the Attitude of $S_{1}$ Is Unchanged

In this case, the navigation problem (19) is simplified as follows: the orbits of both $S_{1}$ and $S_{2}$ are known, and the attitude of $S_{1}$ is unknown but unchanged (i.e., $\omega ={\left[0\;0\;0\right]}^{T}$). Thus, the state vector can be written as:

(33) $$X=q={\left[q_{0},q_{1},q_{2},q_{3}\right]}^{T}$$

The state model is given by:

(34) $$\dot{X}=F\left(X\right)=\dot{q}=g_{1}\left(q\right)=\frac{1}{2}\Omega \left(\omega \right)q=\frac{1}{2}\left[\begin{array}{cc} 0 & -\omega^{T}\\ \omega & -\omega^{\times } \end{array}\right]q={\left[0,0,0,0\right]}^{T}$$

The measurement is shown in Equation (17) and then the partial derivative of the intersatellite angle to the quaternion of spacecraft $S_{1}$$H_{k}$ is of the form:

(35) $$\begin{array}{cc} \; & H_{k}={\left.\frac{\partial y}{\partial q}\right|}_{t=t_{k}}={\left.\frac{\partial R_{E}^{B}}{\partial q}\right|}_{t=t_{k}}\frac{r_{2}-r_{1}}{∥r_{2}-r_{1}∥}\\ \; & =\left[{\left.\frac{\partial R_{E}^{B}}{\partial q_{0}}\right|}_{t=t_{k}}\;{\left.\frac{\partial R_{E}^{B}}{\partial q_{1}}\right|}_{t=t_{k}}\;{\left.\frac{\partial R_{E}^{B}}{\partial q_{2}}\right|}_{t=t_{k}}\;{\left.\frac{\partial R_{E}^{B}}{\partial q_{3}}\right|}_{t=t_{k}}\right]\frac{r_{2}-r_{1}}{∥r_{2}-r_{1}∥} \end{array}$$

For the state model in Equation (34), the Jacobi matrix is given as:

(36) $$\frac{\partial F(X)}{\partial X}=\frac{\partial F(q)}{\partial q}=0_{4\times 4}$$

Therefore, the STM has the following format:

(37) $$\Phi \left(t_{k},t_{0}\right)=I_{4\times 4}$$

### 3.2. Case II: The Orbit of $S_{1}$ Is Known, and the Attitude of $S_{1}$ Is Unchanged

In this case, the orbit of $S_{1}$ is known, and the attitude of $S_{1}$ is unknown but unchanged; hence, the state to be estimated is given by:

(38) $$\dot{X}=F\left(X\right)={\left[{\dot{X}}_{2}^{T},{\dot{q}}^{T}\right]}^{T}={\left[f{\left(X_{2}\right)}^{T},g_{1}{\left(q\right)}^{T}\right]}^{T}={\left[f{\left(X_{2}\right)}^{T},\left[0,0,0,0\right]\right]}^{T}$$

The corresponding state model is as follows:

(39) $$\dot{X}=F\left(X\right)={\left[{\dot{X}}_{2}^{T},{\dot{q}}^{T}\right]}^{T}={\left[f{\left(X_{2}\right)}^{T},g_{1}{\left(q\right)}^{T}\right]}^{T}={\left[f{\left(X_{2}\right)}^{T},\left[0,0,0,0\right]\right]}^{T}$$

The partial differential matrix $H_{k}$ of measurement (17) is written as:

(40) $$\begin{array}{cc} \; & H_{k}={\left.\frac{\partial y}{\partial X}\right|}_{t=t_{k}}=\left[{\left.\frac{\partial y}{\partial X_{2}}\right|}_{t=t_{k}}\;{\left.\frac{\partial y}{\partial q}\right|}_{t=t_{k}}\right]=\left[{\left.\frac{\partial y}{\partial r_{2}}\right|}_{t=t_{k}}\;{\left.\frac{\partial y}{\partial v_{2}}\right|}_{t=t_{k}}\;{\left.\frac{\partial y}{\partial q}\right|}_{t=t_{k}}\right]\\ \; & =\left[R_{E}^{B}{\left.\frac{\partial }{\partial r_{2}}\left(\frac{r_{2}-r_{1}}{∥r_{2}-r_{1}∥}\right)\right|}_{t=t_{k}}\;R_{E}^{B}{\left.\frac{\partial }{\partial v_{2}}\left(\frac{r_{2}-r_{1}}{∥r_{2}-r_{1}∥}\right)\right|}_{t=t_{k}}\;{\left.\frac{\partial R_{E}^{B}}{\partial q}\right|}_{t=t_{k}}\frac{r_{2}-r_{1}}{∥r_{2}-r_{1}∥}\right] \end{array}$$

The propagation of the STM for state model (39) is then given by:

(41) $$\left\{\begin{array}{c} \Phi \left(t_{0},t_{0}\right)=I_{10\times 10}\\ \dot{\Phi }\left(t_{k},t_{0}\right)={\left.\frac{\partial F(X)}{\partial X}\right|}_{t=t_{k}}\Phi \left(t_{k},t_{0}\right) \end{array}\right.$$

Hence, the STM $\Phi (t_{k},t_{0})$ has the following format:

(42) $$\Phi \left(t_{k},t_{0}\right)=\left[\begin{array}{ccc} \Phi_{rr}\left(t_{k},t_{0}\right) & \Phi_{rv}\left(t_{k},t_{0}\right) & 0_{3\times 4}\\ \Phi_{vr}\left(t_{k},t_{0}\right) & \Phi_{vv}\left(t_{k},t_{0}\right) & 0_{3\times 4}\\ 0_{4\times 3} & 0_{4\times 3} & I_{4\times 4} \end{array}\right]$$

### 3.3. Case III: Both the Orbits of $S_{1}$ and $S_{2}$ Are Unknown, and the Attitude of $S_{1}$ Is Unchanged

In this case, the orbits of $S_{1}$ and $S_{2}$ as well as the attitude of $S_{1}$ are estimated. The only information for the navigation system is that $\omega ={\left[0\;0\;0\right]}^{T}$. Therefore, the state to be estimated is:

(43) $$X={\left[X_{1}^{T},X_{2}^{T},q^{T}\right]}^{T}={\left[x_{1},y_{1},z_{1},{\dot{x}}_{1},{\dot{y}}_{1},{\dot{z}}_{1},x_{2},y_{2},z_{2},{\dot{x}}_{2},{\dot{y}}_{2},{\dot{z}}_{2},q_{0},q_{1},q_{2},q_{3}\right]}^{T}$$

The state model is then given by:

(44) $$\dot{X}=F\left(X\right)={\left[{\dot{X}}_{1}^{T},{\dot{X}}_{2}^{T},{\dot{q}}^{T}\right]}^{T}={\left[f{\left(X_{1}\right)}^{T},f{\left(X_{1}\right)}^{T},g_{1}{\left(q\right)}^{T}\right]}^{T}={\left[f{\left(X_{1}\right)}^{T},f{\left(X_{1}\right)}^{T},\left[0,0,0,0\right]\right]}^{T}$$

Similar to Equation (40), the partial differential matrix of measurement is given as:

(45) $$\begin{array}{cc} \; & H_{k}={\left.\frac{\partial y}{\partial X}\right|}_{t=t_{k}}=\left[{\left.\frac{\partial y}{\partial X_{1}}\right|}_{t=t_{k}}\;{\left.\frac{\partial y}{\partial X_{2}}\right|}_{t=t_{k}}\;{\left.\frac{\partial y}{\partial q}\right|}_{t=t_{k}}\right]=\left[{\left.\frac{\partial y}{\partial r_{1}}\right|}_{t=t_{k}}\;{\left.\frac{\partial y}{\partial v_{1}}\right|}_{t=t_{k}}\;{\left.\frac{\partial y}{\partial r_{2}}\right|}_{t=t_{k}}\;{\left.\frac{\partial y}{\partial v_{2}}\right|}_{t=t_{k}}\;{\left.\frac{\partial y}{\partial q}\right|}_{t=t_{k}}\right]\\ \; & ={\left.\left[R_{E}^{B}\frac{\partial }{\partial r_{1}}\left(\frac{r_{2}-r_{1}}{∥r_{2}-r_{1}∥}\right),\;R_{E}^{B}\frac{\partial }{\partial v_{1}}\left(\frac{r_{2}-r_{1}}{∥r_{2}-r_{1}∥}\right),\;R_{E}^{B}\frac{\partial }{\partial r_{2}}\left(\frac{r_{2}-r_{1}}{∥r_{2}-r_{1}∥}\right),\;R_{E}^{B}\frac{\partial }{\partial v_{2}}\left(\frac{r_{2}-r_{1}}{∥r_{2}-r_{1}∥}\right),\;\frac{\partial R_{E}^{B}}{\partial q}\frac{r_{2}-r_{1}}{∥r_{2}-r_{1}∥}\right]\right|}_{t=t_{k}} \end{array}$$

where:

(46) $$\frac{\partial }{\partial r_{1}}\left(\frac{r_{2}-r_{1}}{∥r_{2}-r_{1}∥}\right)=-\frac{1}{∥r_{2}-r_{1}∥}\left[I_{3\times 3}-\frac{\left(r_{2}-r_{1}\right){\left(r_{2}-r_{1}\right)}^{T}}{{∥r_{2}-r_{1}∥}^{2}}\right]$$

(47) $$\frac{\partial }{\partial v_{1}}\left(\frac{r_{2}-r_{1}}{∥r_{2}-r_{1}∥}\right)=0_{3\times 3}$$

The STM $\Phi (t_{k},t_{0})$ in this case has the following format:

(48) $$\Phi \left(t_{k},t_{0}\right)=\left[\begin{array}{ccccc} \Phi_{r_{1}r_{1}}\left(t_{k},t_{0}\right) & \Phi_{r_{1}v_{1}}\left(t_{k},t_{0}\right) & 0_{3\times 3} & 0_{3\times 3} & 0_{3\times 4}\\ \Phi_{v_{1}r_{1}}\left(t_{k},t_{0}\right) & \Phi_{v_{1}v_{1}}\left(t_{k},t_{0}\right) & 0_{3\times 3} & 0_{3\times 3} & 0_{3\times 4}\\ 0_{3\times 3} & 0_{3\times 3} & \Phi_{r_{2}r_{2}}\left(t_{k},t_{0}\right) & \Phi_{r_{2}v_{2}}\left(t_{k},t_{0}\right) & 0_{3\times 4}\\ 0_{3\times 3} & 0_{3\times 3} & \Phi_{v_{2}r_{2}}\left(t_{k},t_{0}\right) & \Phi_{v_{2}v_{2}}\left(t_{k},t_{0}\right) & 0_{3\times 4}\\ 0_{4\times 3} & 0_{4\times 3} & 0_{4\times 3} & 0_{4\times 3} & I_{4\times 4} \end{array}\right]$$

### 3.4. Case IV: Both the Orbits of $S_{1}$ and $S_{2}$ Are Unknown, and the Angular Velocity of $S_{1}$ Is Known

In this case, the attitude of $S_{1}$ is unknown and changed. For spacecraft $S_{1}$, the angular velocity $\omega ={\left[\omega_{x}\;\omega_{y}\;\omega_{z}\right]}^{T}$ is foreknown (assume that $\omega_{x}$, $\omega_{y}$ and $\;\omega_{z}$ are constant and satisfy $\omega_{x}^{2}+\omega_{y}^{2}+\omega_{z}^{2}\neq 0$). Hence, the state vector to be estimated is the same as that of case III, with the form of Equation (43). The state model is written as:

(49) $$\dot{X}=F\left(X\right)={\left[{\dot{X}}_{1}^{T},{\dot{X}}_{2}^{T},{\dot{q}}^{T}\right]}^{T}={\left[f{\left(X_{1}\right)}^{T},f{\left(X_{1}\right)}^{T},g_{1}{\left(q\right)}^{T}\right]}^{T}={\left[f{\left(X_{1}\right)}^{T},f{\left(X_{1}\right)}^{T},q^{T}\Omega^{T}\left(\omega \right)/2\;\right]}^{T}$$

The STM is in the form of:

(50) $$\Phi \left(t_{k},t_{0}\right)=\left[\begin{array}{ccccc} \Phi_{r_{1}r_{1}}\left(t_{k},t_{0}\right) & \Phi_{r_{1}v_{1}}\left(t_{k},t_{0}\right) & 0_{3\times 3} & 0_{3\times 3} & 0_{3\times 4}\\ \Phi_{v_{1}r_{1}}\left(t_{k},t_{0}\right) & \Phi_{v_{1}v_{1}}\left(t_{k},t_{0}\right) & 0_{3\times 3} & 0_{3\times 3} & 0_{3\times 4}\\ 0_{3\times 3} & 0_{3\times 3} & \Phi_{r_{2}r_{2}}\left(t_{k},t_{0}\right) & \Phi_{r_{2}v_{2}}\left(t_{k},t_{0}\right) & 0_{3\times 4}\\ 0_{3\times 3} & 0_{3\times 3} & \Phi_{v_{2}r_{2}}\left(t_{k},t_{0}\right) & \Phi_{v_{2}v_{2}}\left(t_{k},t_{0}\right) & 0_{3\times 4}\\ 0_{4\times 3} & 0_{4\times 3} & 0_{4\times 3} & 0_{4\times 3} & e^{\frac{\Omega (\omega )}{2}} \end{array}\right]$$

### 3.5. Case V: Both the Orbits of $S_{1}$ and $S_{2}$ Are Unknown, and the Angular Velocity of $S_{1}$ Is Unchanged

In this case, the angular velocity $\omega ={\left[\omega_{x}\;\omega_{y}\;\omega_{z}\right]}^{T}$ also exists, unknown but unchanged. Therefore, the state vector is obtained by Equation (13) and the state model is given as Equation (14).

The partial derivative of the intersatellite angle to the state vector X is as follows:

(51) $$\begin{array}{cc} H_{k}= & {\left.\frac{\partial y}{\partial X}\right|}_{t=t_{k}}=\left[{\left.\frac{\partial y}{\partial X_{1}}\right|}_{t=t_{k}}\;{\left.\frac{\partial y}{\partial X_{2}}\right|}_{t=t_{k}}\;{\left.\frac{\partial y}{\partial q}\right|}_{t=t_{k}}\;{\left.\frac{\partial y}{\partial \omega }\right|}_{t=t_{k}}\right]\\ = & \left[{\left.\frac{\partial y}{\partial r_{1}}\right|}_{t=t_{k}}\;{\left.\frac{\partial y}{\partial v_{1}}\right|}_{t=t_{k}}\;{\left.\frac{\partial y}{\partial r_{2}}\right|}_{t=t_{k}}\;{\left.\frac{\partial y}{\partial v_{2}}\right|}_{t=t_{k}}\;{\left.\frac{\partial y}{\partial q}\right|}_{t=t_{k}}\;{\left.\frac{\partial y}{\partial \omega }\right|}_{t=t_{k}}\right]\\ = & \left[R_{E}^{B}\frac{\partial }{\partial r_{1}}\left(\frac{r_{2}-r_{1}}{∥r_{2}-r_{1}∥}\right),\;R_{E}^{B}\frac{\partial }{\partial v_{1}}\left(\frac{r_{2}-r_{1}}{∥r_{2}-r_{1}∥}\right),\;R_{E}^{B}\frac{\partial }{\partial r_{2}}\left(\frac{r_{2}-r_{1}}{∥r_{2}-r_{1}∥}\right)\right.,\\ & {\left.\left.\;R_{E}^{B}\frac{\partial }{\partial v_{2}}\left(\frac{r_{2}-r_{1}}{∥r_{2}-r_{1}∥}\right),\;\frac{\partial R_{E}^{B}}{\partial q}\frac{r_{2}-r_{1}}{∥r_{2}-r_{1}∥},\frac{\partial R_{E}^{B}}{\partial \omega }\frac{r_{2}-r_{1}}{∥r_{2}-r_{1}∥}\right]\right|}_{t=t_{k}} \end{array}$$

## 4. Numerical Simulation

In this section, a series of numerical results for several types of scenarios is presented, with the following three objectives: (1) to demonstrate whether the system is observable or unobservable for each scenario and (2) to validate the observability indices by comparing their estimations to the quality of the solution of the state estimation problem using the fifth-order CKF.

### 4.1. Simulation Background

**Figure 2:**
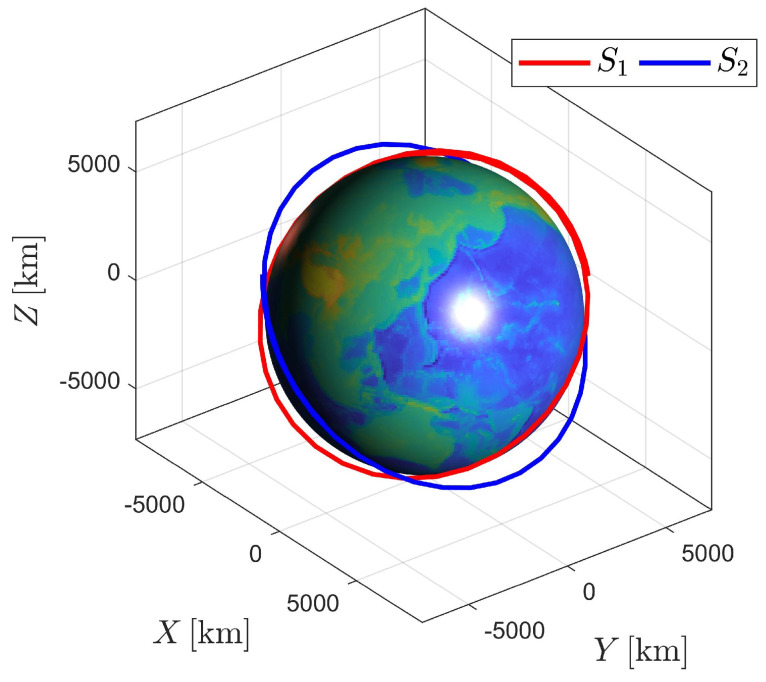
Orbits of spacecraft $S_{1}$ and $S_{2}$.

An example with two spacecraft in circle orbits is considered. The nominal orbit elements are listed in Table 1, and the corresponding orbits are shown in Figure 2. The elements *h*, *e*, *i*, Ω, ω, and *n* denote the orbit altitude, eccentricity, inclination, longitude of the ascending node, and argument of the periapse and true anomaly, respectively. The semimajor axis a is computed by $a=h+R_{e}$, where $R_{e}=6378.137\;\mathrm{km}$ is the radius of the Earth.

The initial position and velocity errors (if unknown) are set to 10 km and 10${}^{-3}$ km/s, respectively. The initial attitude of spacecraft $S_{1}$ is set to be $q_{13}={\left[0,0,0\right]}^{T}$ and $q_{0}=\sqrt{1-q_{13}^{T}q_{13}}=1$. For all the cases above, the initial estimation of the quaternion is given by:

(52) $$\hat{q}={\left[\cos\frac{\Theta }{2}\;\frac{\sqrt{3}}{3}\sin\frac{\Theta }{2}\;\frac{\sqrt{3}}{3}\sin\frac{\Theta }{2}\;\frac{\sqrt{3}}{3}\sin\frac{\Theta }{2}\right]}^{T}$$

where $\Theta =5^{\circ }$.

The initial angular velocity of spacecraft $S_{1}$, if existing (i.e., for case IV and case V), is set to be $\omega ={\left[0.01,0.01,0.01\right]}^{T}\;{}^{\circ }/s\;$. The initial estimation of angular velocity is considered as $\hat{\omega }={\left[0.09,0.09,0.09\right]}^{T}\;{}^{\circ }/s\;$ if ω is to be estimated (only for case V).

The initial covariance matrix $P_{0}$ is given as $P_{r_{1}r_{1},0}=P_{r_{2}r_{2},0}=100I_{3\times 3}\;km^{2}$, $P_{v_{1}v_{1},0}=P_{v_{2}v_{2},0}=I_{3\times 3}\;{m/s\;}^{2}$, $P_{q_{0},0}={\left(\cos2.5^{\circ }-1\right)}^{2}I_{1\times 1}$, $P_{q_{1},0}=P_{q_{2},0}=P_{q_{3},0}={\left(\sin2.5^{\circ }/\sqrt{3}\;\right)}^{2}I_{1\times 1}$ and $P_{\omega ,0}=0.001^{2}I_{3\times 3}\;({}^{\circ }/s\;{)}^{2}$.

Suppose spacecraft $S_{1}$ could track $S_{2}$ using optical equipment, where possible, with a 1 s measurement. The angle-measurement error in Equation (17) is assumed to be Gaussian white noise with a standard deviation (STD) of $0.01^{\circ }$ (equal to $0.6^{\prime }$).

### 4.2. Results and Discussion

During the observability test, the state transfer matrix is obtained using MATLAB function ode45, the rank of OM rank(N) is calculated using function rank and the CN of the system cond(N) is obtained by function cond. All the following operations are executed on MATLAB R2018b [40]. In addition, the particular situations for the autonomous attitude and orbit determination with two spacecraft are simulated to verify the observability analysis. The nominal orbit elements are given in Table 1. The estimation problems are solved using the traditional fifth-order CKF. For convenience, the quaternion of spacecraft $S_{1}$ is transferred into the form of Euler angles using the MATLAB function quat2angle. The results are given as follows.

#### 4.2.1. Case I: Both Orbits of $S_{1}$ and $S_{2}$ Are Known, and the Attitude of $S_{1}$ Is Unchanged

**Figure 3:**
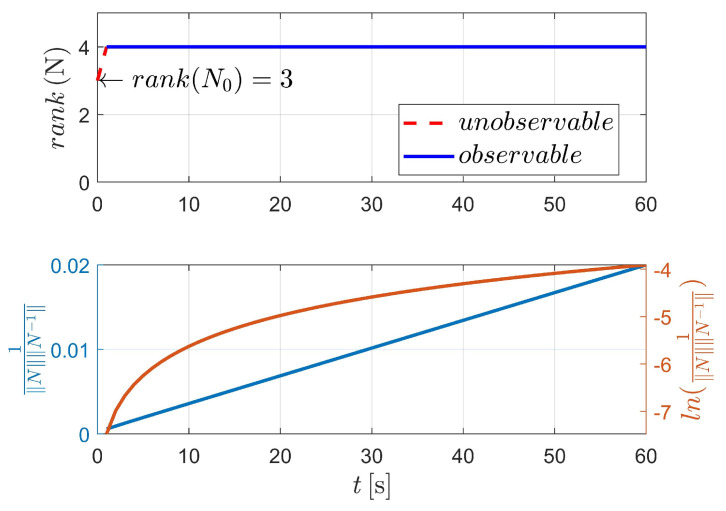
Observability simulation of case I (nonperturbation).

Figure 3 displays the observability results of case I, using the two-body dynamics (state model as Equation (3)). For case I, 60 measurements (equivalent to 60 s) are executed during the navigation process. As shown in Figure 3, the upper stacked subplot illustrates the rank of the observability matrix (OM), where the red line represents the unobservable period (i.e., rank(N)<4) and the blue line represents the observable period (i.e., rank(N)≥4). Moreover, the lower stacked subplot demonstrates the condition number (CN) of OM (note that in Figure 3, the y-label represents the reciprocal of CN). In addition, the logarithmic value of the reciprocal of CN is also given in the lower stacked subplot, making the change curve of CN more obvious. Figure 3 shows that the CN deceases with respect to the observation time, which means that the observability of the system continuously improves as the number of measurements increases.

The detailed value of the corresponding index in Figure 3 is selectively illustrated in Table 2. For case I, the system is completely observable after only two measurements (at epochs $t_{0}$ and $t_{k}$, respectively).

**Figure 4:**
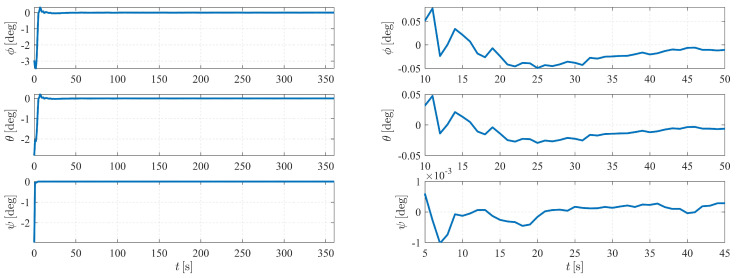
Attitude determination errors of case I (nonperturbation) and the corresponding larger plot.

Figure 4 depicts the estimation errors of the attitude of spacecraft $S_{1}$. It is shown that the errors converge to zero at approximately 50 s when the initial Euler angle errors are set to be $2.5^{\circ }$.

#### 4.2.2. Case II: The Orbit of $S_{1}$ Is Known, and the Attitude of $S_{1}$ Is Unchanged

**Figure 5:**
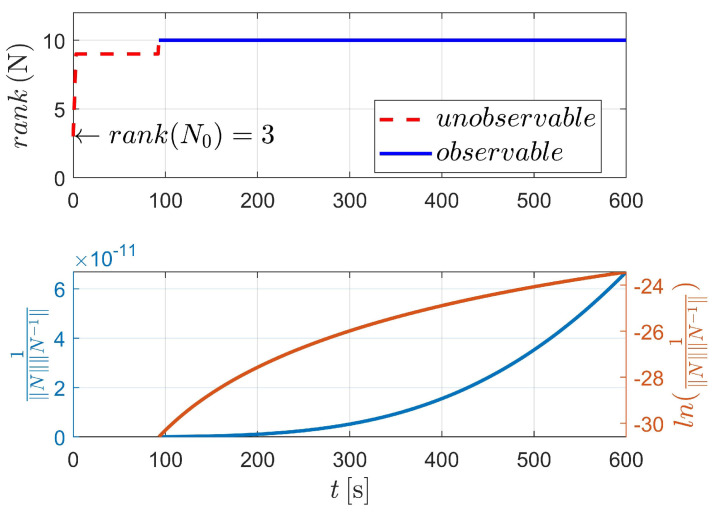
Observability simulation of case II (nonperturbation).

The observability results of case II are illustrated in Figure 5 and Table 3. As indicated in Figure 4, the system of Equation (39) is observable after approximately 94 measurements (from epoch $t_{0}$ to epoch $t_{93}$). Compared with case I, the system corresponding to case II is much more difficult to observe. Note that as containing the state vector of target spacecraft $S_{2}$, the dimension of the state to be estimated in case II is higher than that of case I, which implies more effort in measurements.

**Figure 6:**
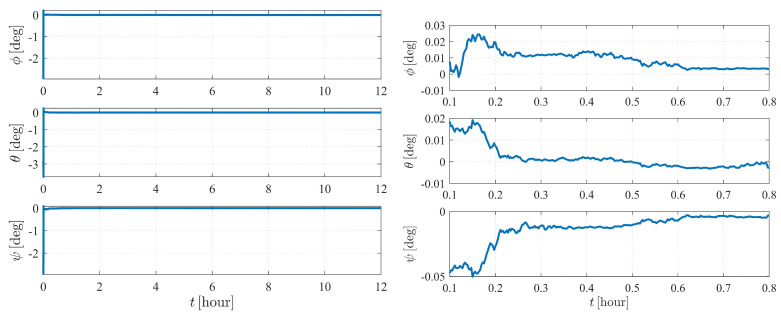
Attitude determination errors of case II (nonperturbation) and the corresponding larger plot.

**Figure 7:**
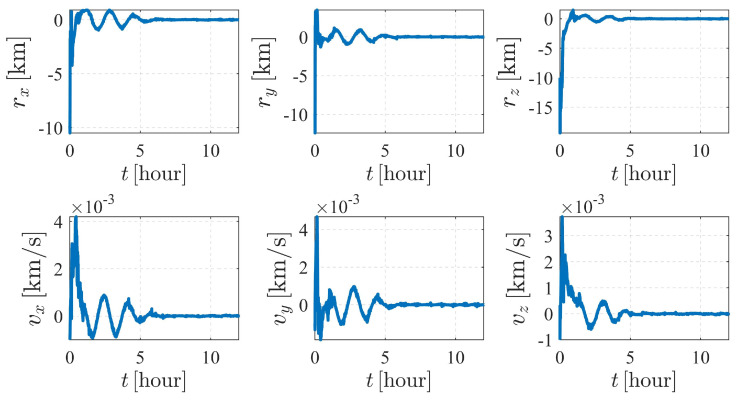
Orbit determination errors of spacecraft $S_{2}$ in case II (nonperturbation).

The estimation results are illustrated in Figure 6 and Figure 7. As shown in Figure 6, the attitude converges much faster than the orbit state, as the previous item converges within only a few minutes, while it takes approximately five hours for the orbit state of spacecraft $S_{2}$ to converge. Note that measurement model (17) is much more sensitive to the attitude of observer $S_{1}$ than the orbits of the observer and the target. Therefore, the attitude converges before the orbit state converges. Note that the observation interval has no effect on the observability, so the influence of shadow or light conditions are not considered in this paper. In most cases, the long-term observation in Figure 6 is impossible to realize due to eclipses, but this simplification is reasonable considering the research content of this paper.

#### 4.2.3. Case III: Both the Orbits of $S_{1}$ and $S_{2}$ Are Unknown, and the Attitude of $S_{1}$ Is Unchanged

**Figure 8:**
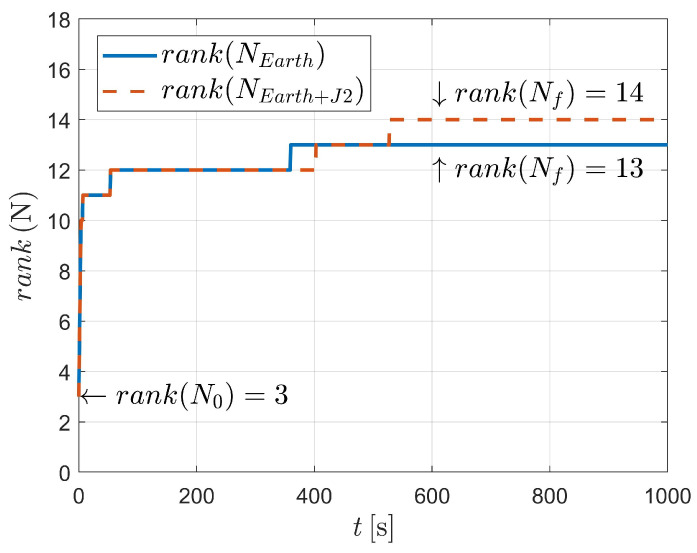
Observability simulation of case III (nonperturbation and with $J_{2}$ perturbation).

**Figure 9:**
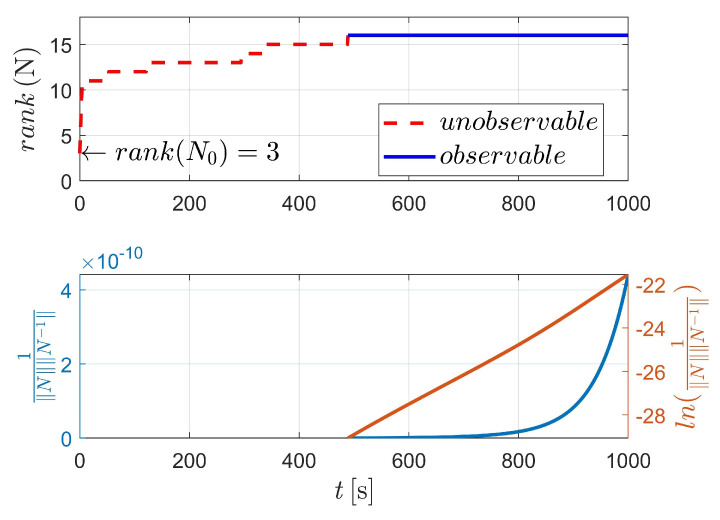
Observability simulation of case III (with $J_{2}$, $J_{3}$, and $J_{4}$ perturbations).

In this case, three kinds of dynamics (state model as Equation (3) and Equation (4), respectively) are considered, as illustrated in Figure 8 and Figure 9. It can be concluded that for dynamics with nonperturbation and $J_{2}$ perturbation, the systems are unobservable (as shown in Figure 8).

For case III, the state vector with 16 variables is estimated. However, when considering two-body dynamics, the observable states are 13-dimensional, which means that only 13 variables (or variable combinations) of the 16-dimensional state vector can be observed. In addition, when taking the $J_{2}$ perturbation into consideration, one more state variable (or variable combination) could be observed, suggesting that the system is still unobserved.

Fortunately, as depicted in Figure 6 and Table 4, the system is observable when $J_{2}$, $J_{3}$ and $J_{4}$ perturbations are considered. In this situation, a total of 16 variables are observed after 490 angle-only measurements. It should be noted that strictly two-body dynamics, or dynamics with a particular perturbation, are, of course, unlikely. This is because aspheric perturbation of the celestial body (i.e., Earth in this paper) contains higher order terms. Moreover, the solar pressure, atmospheric drag and gravitational perturbation of the third body could also influence the orbiting spacecraft. However, the significance of the observability analysis is to state (as might be expected) that when the dynamics are very close to the two-body dynamics (e.g., when an orbit is high, for example, high-orbit GPS satellites), it is difficult to estimate the orbit because the influence of aspheric perturbation is weak.

**Figure 10:**
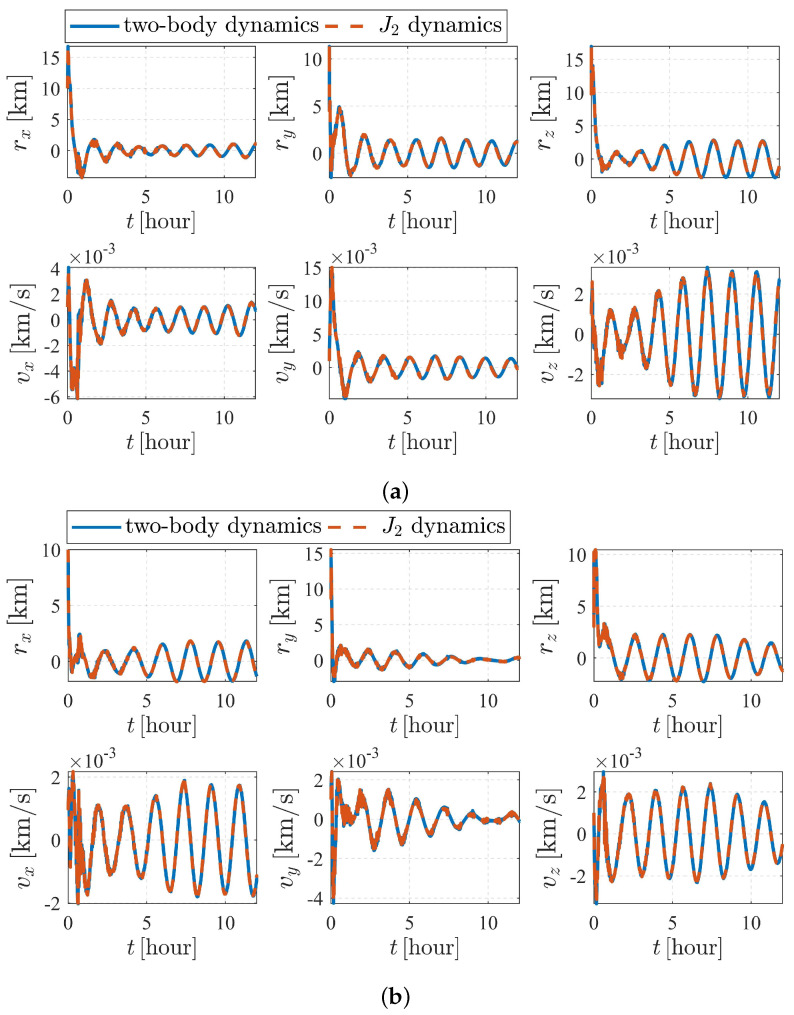
Orbit determination errors of case III (nonperturbation and with $J_{2}$ perturbation). (**a**) Orbit estimation errors of spacecraft $S_{1}$. (**b**) Orbit estimation errors of spacecraft $S_{2}$.

The simulation results of the subcase with nonperturbation and $J_{2}$ perturbation are illustrated in Figure 10. It was shown that estimation quality is poor and appears to be diverging (the $v_{z}$ for $S_{1}$ and $v_{x}$ for $S_{2}$ are clearly divergent with time), meaning that the system is unobservable under the given dynamics and initial conditions, which validates the observability analysis listed in Table 4.

**Figure 11:**
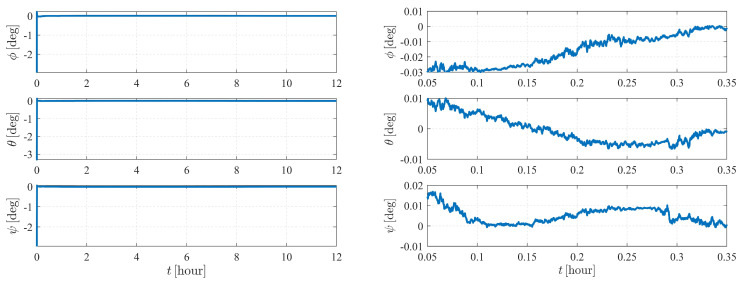
Attitude determination errors of case III (with $J_{2}$, $J_{3}$, and $J_{4}$ perturbations) and the corresponding larger plot.

**Figure 12:**
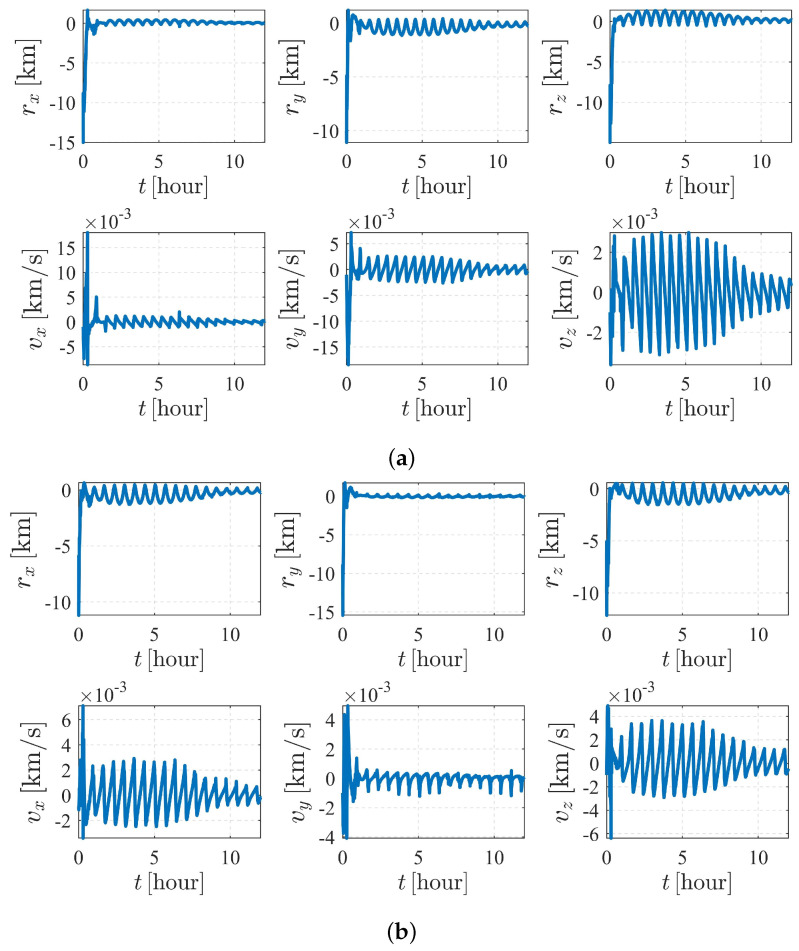
Orbit determination errors of case III (with $J_{2}$, $J_{3}$, and $J_{4}$ perturbations. (**a**) Orbit estimation errors of spacecraft $S_{1}$. (**b**) Orbit estimation errors of spacecraft $S_{2}$.

Figure 11 and Figure 12 depict the autonomous attitude and orbit determination results of the two spacecraft considering $J_{2}$, $J_{3}$, and $J_{4}$ perturbations. Although exhibiting an obvious oscillation, the system still succeeds in converging. However, compared with the results in case II, the convergence is much weaker, meaning that this form is not stable enough. It is noted that although we have proven that case II under dynamics (5) is observable, this does not contradict the estimation results obtained here but implies that the system is higher-order locally weakly observable [24].

#### 4.2.4. Case IV: Both the Orbits of $S_{1}$ and $S_{2}$ Are Unknown, and the Angular Velocity of $S_{1}$ Is Known

**Figure 13:**
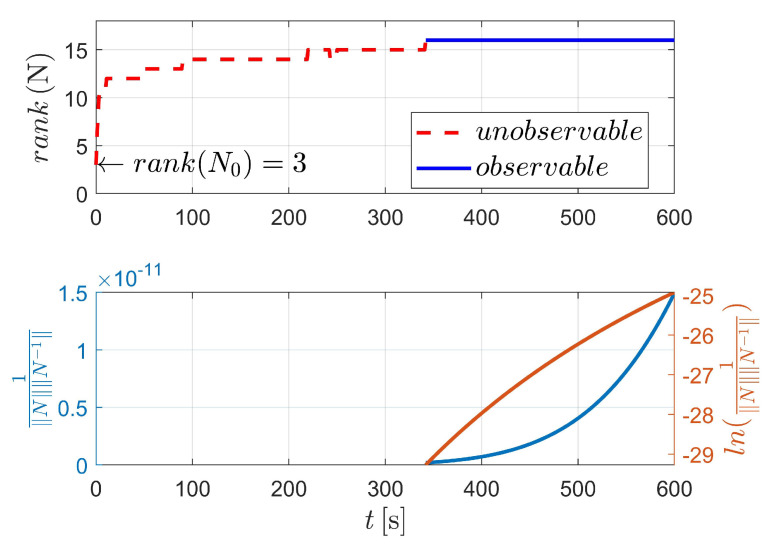
Observability simulation of case IV (nonperturbation).

**Figure 14:**
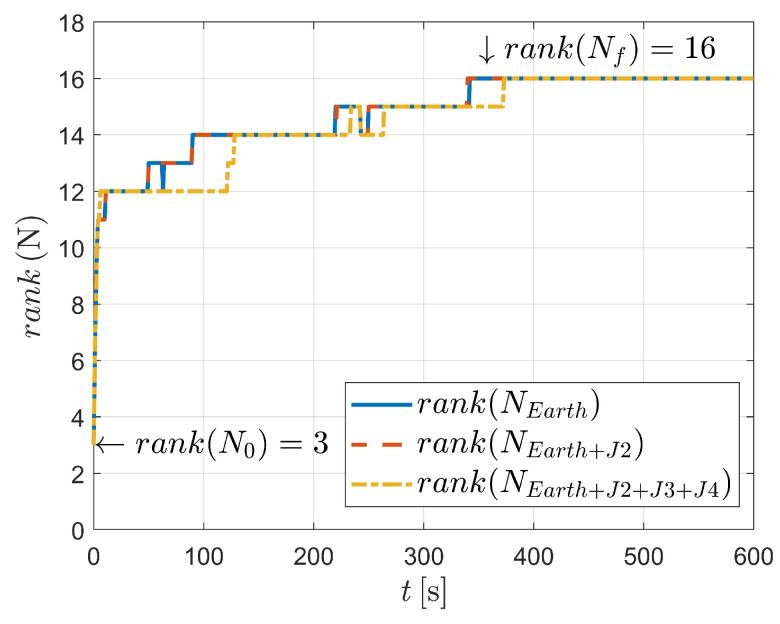
Observability comparisons of case IV.

In case IV, the results are demonstrated in Figure 13 and Figure 14 and Table 5. As shown in Figure 13 and Table 5, when applying a known attitude maneuver to observer spacecraft $S_{1}$, the system is completely observable even under the simplest two-body dynamics.

As indicated in Table 5, only 344 measurements are needed to observe the 16 variables, which is less than that of case III (situation considering $J_{2}$, $J_{3}$, and $J_{4}$ perturbations in Table 4). Furthermore, at time epoch $t_{489}$, which is difficult to observe in case III, the CN of the system is larger than that of case IV (for case III, as shown in Table 4, $\frac{1}{\mathrm{cond}(N)}=2.3821\times 10^{-13}$, while for case IV, listed in Table 5, $\frac{1}{\mathrm{cond}(N)}=3.4291\times 10^{-12}$), indicating that compared to the perturbation acceleration, a suitable attitude maneuver is more likely to attach obvious improvement to the observability of the two-spacecraft system.

Figure 8 compares the influence of different dynamics. In Figure 14, the blue solid line, the red dashed line and the orange dash-dotted line represent the dynamics (3) and (4), respectively. It is illustrated that, for all three conditions, the system becomes observable around epoch $t_{350}$, which means that the dynamics make no difference to the observability of the system (note that the subtle difference could be recognized as the outcome of numerical calculation during the usage of ode45, rank and cond).

**Figure 15:**
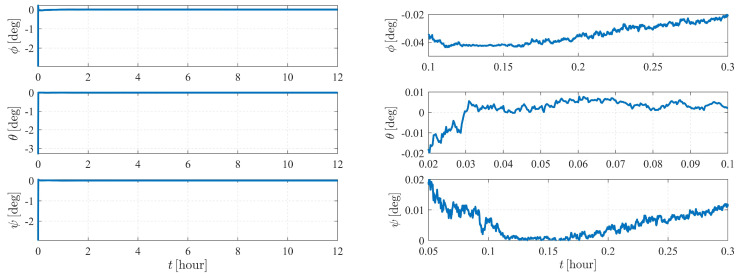
Attitude determination errors of case IV (nonperturbation) and the corresponding larger plot.

**Figure 16:**
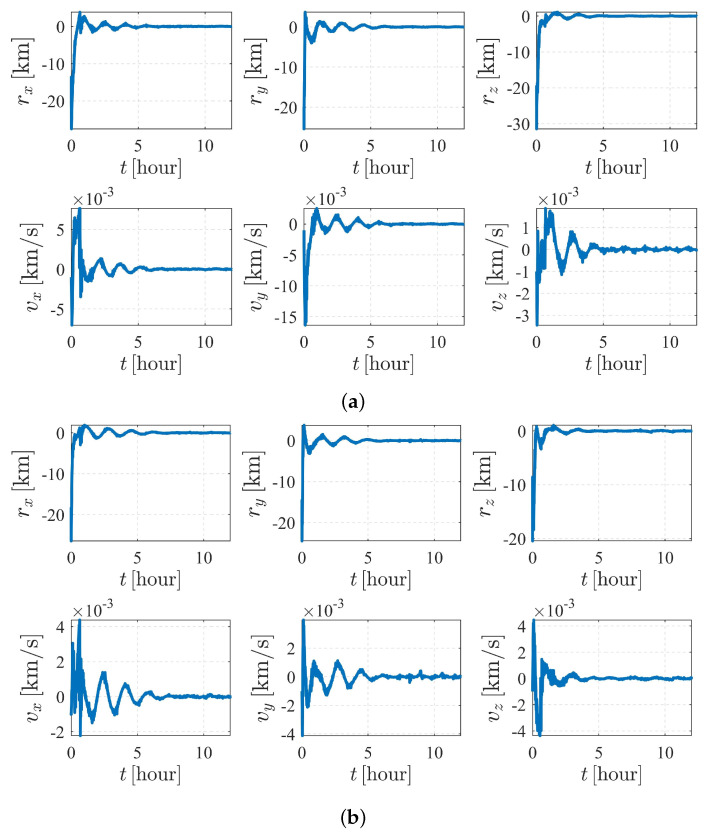
Orbit determination errors of case IV (nonperturbation). (**a**) Orbit estimation errors of spacecraft $S_{1}$. (**b**) Orbit estimation errors of spacecraft $S_{2}$.

The autonomous attitude and orbit determination results of case IV are shown in Figure 15 and Figure 16, respectively. The results show strong convergence within five hours, implying that the corresponding system is completely observable. In addition, it is observed that the state estimations of Case IV present a significantly better stability than those of Case III. With a known attitude maneuver executed, the navigation system is more stable, and the certainty of the estimates improves compared with the results shown in Figure 12. In conclusion, by comparison, the attitude maneuver makes the system more observable and the estimation more accurate.

#### 4.2.5. Case V: Both the Orbits of $S_{1}$ and $S_{2}$ Are Unknown, and the Angular Velocity of $S_{1}$ Is Unchanged

**Figure 17:**
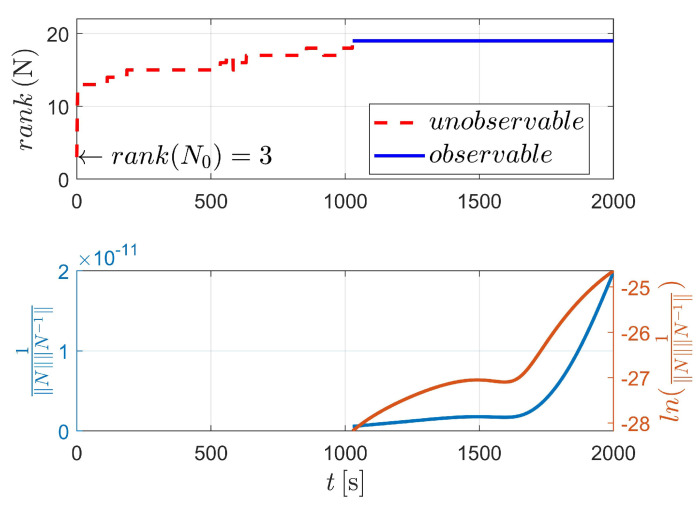
Observability simulation of case V (with nonperturbation).

The test results of case V are illustrated in Figure 17 and Table 6. When not considering any perturbation, the system is observable at epoch $t_{1028}$. Compared with the situation in which the angular velocity of $S_{1}$ is known, it is slightly more difficult to estimate the system when the angular velocity needs to be determined.

**Figure 18:**
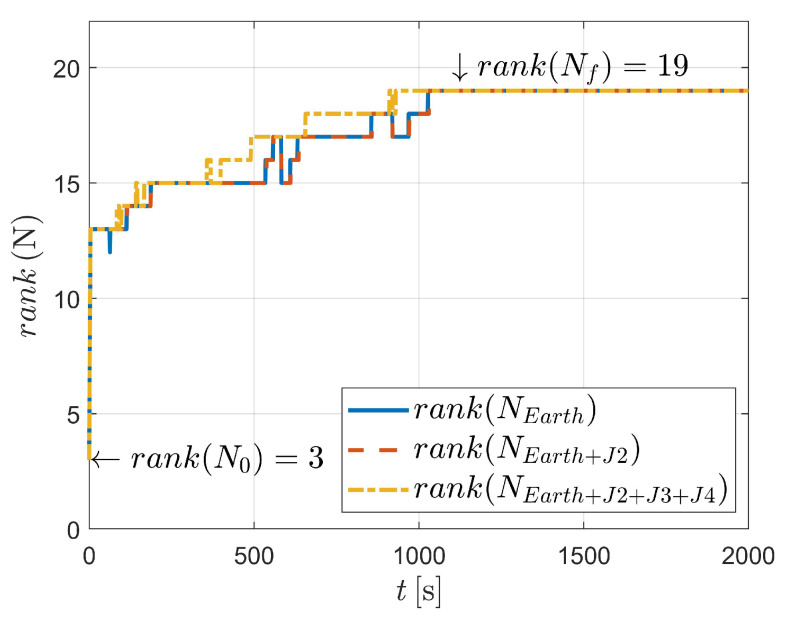
Observability comparisons of case V.

As shown in Figure 18, the conclusions are summarized that the perturbations have almost no influence on the observability of the system, although the angular velocity is unknown.

**Figure 19:**
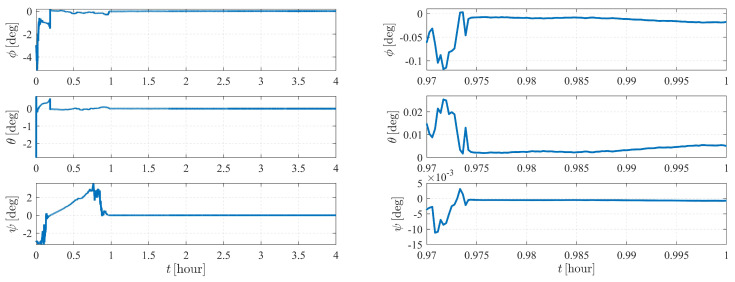
Attitude determination errors of case V (nonperturbation) and the corresponding larger plot.

**Figure 20:**
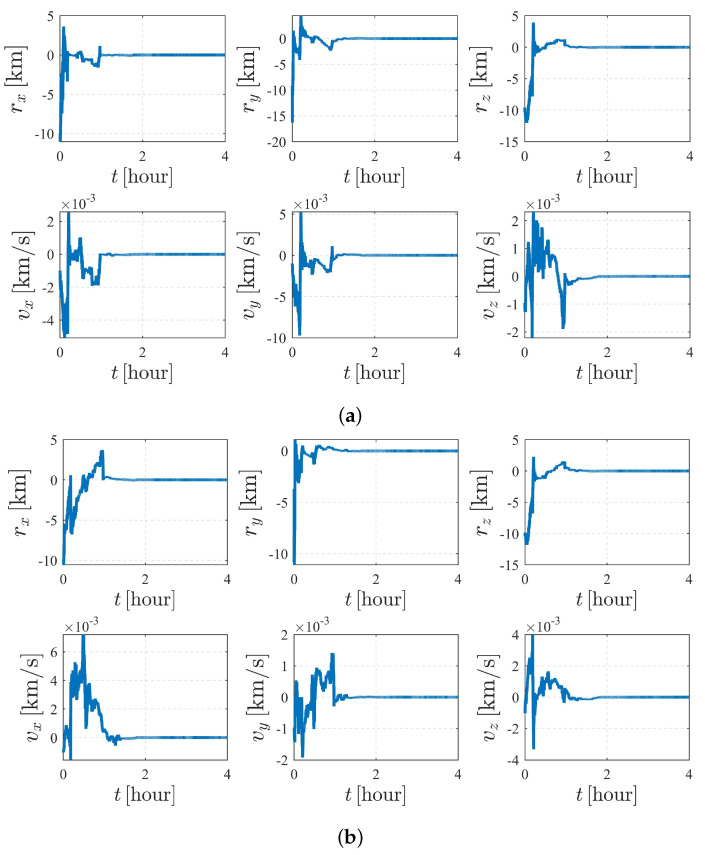
Orbit determination errors of case V (nonperturbation). (**a**) Orbit estimation errors of spacecraft $S_{1}$. (**b**) Orbit estimation errors of spacecraft $S_{2}$.

**Figure 21:**
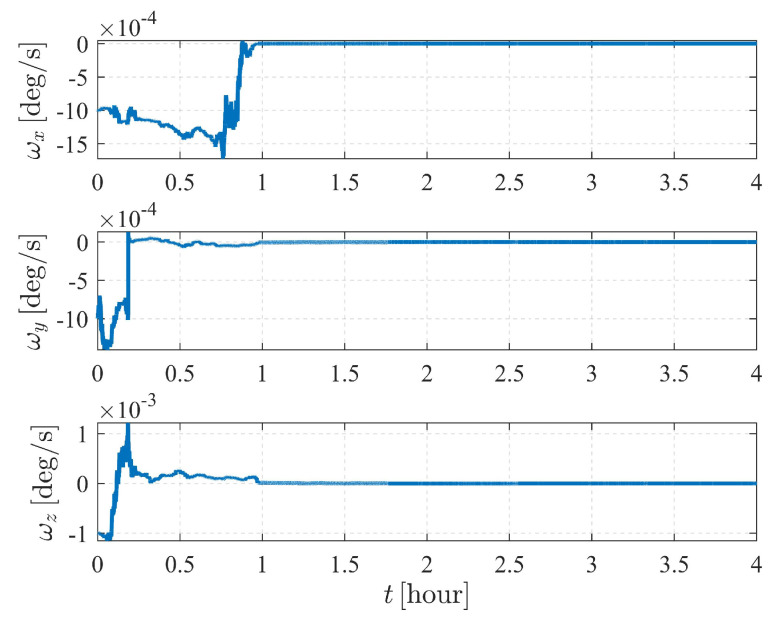
The estimation errors of angular velocity of case V (nonperturbation).

Note that the system is sensitive to the attitude and the angular velocity of $S_{1}$; hence, for case V, the numerical observability measures are computed from the initial simulation epoch to the end of the simulation (within a total of 4 h) with a measurement frequency of 1 per 0.5 s. The results are illustrated in Figure 19, Figure 20 and Figure 21. Figure 19 and Figure 21 show that the attitude and the angular velocity converge at approximately 1 h, while as expected, the orbit states converge at approximately 2 h (Figure 20).

Even under the situation in which the angular velocity is to be estimated, the estimation quality of case V is still healthier than that of case III, indicating that the attitude maneuver is superior to the complex perturbation with respect to system observability.

The above discussion of the observability of the attitude and orbit is summarized in Table 7. Note that in Table 7, the symbol ‘**✓**’ denotes that the corresponding item is known, while the symbol ‘**✗**’ states that the item is unknown. Moreover, one item is suggested to not exist if it is marked ‘*none*’. For example, subcase II of case III indicates that the $J_{2}$ perturbation is considered, both the orbits of $S_{1}$ and $S_{2}$ and the attitude of $S_{1}$ are estimated, and no attitude maneuver is executed.

## 5. Conclusions

In this paper, the autonomous attitude and orbit determination problem of a two-spacecraft system using angle-only measurements is studied. The observability of the system is analyzed based on the theory of observability matrix. Five cases are analyzed and the observability analysis results are as follows:

• When the orbits of both observer and target are known, and the attitude of the target is unknown and unchanged, the navigation system is observable.
• When the orbit of observer is known, and the attitude and orbit of the target are unknown, the navigation system is observable.
• When the orbits of observer and target and the attitude of the target are unknown, the navigation system is unobservable in the two-body dynamics. The navigation system becomes observable when considering high-order perturbations.
• When the orbits of observer and target and the attitude of the target are unknown, and the attitude of the target is changed, the navigation system is observable.

In addition, the observability analysis and the filter results both verify that compared to the perturbation acceleration, a suitable attitude maneuver is more likely to attach obvious improvement to the observability of the two-spacecraft system. 

## Figures and Tables

**Table 1:** Nominal orbit elements of the circle orbits.

Spacecraft	*h*/km	*e*	*i*/deg	Ω/deg	ω/deg	*n*/deg
$S_{1}$	500	0	45.05	29.93	132.9	−107.74
$S_{2}$	1000	0	45	94.8	199.0	−54.13

**Table 2:** Observability test results of case I (nonperturbation).

Epoch $t_{k}$	Rank(N)	Observability	1/cond(N)	ln[1/cond(N)]
0	3	False	-	-
1	4	True	0.0005	−7.4740
2	4	True	0.0009	−6.9835
3	4	True	0.0012	−6.6692

**Table 3:** Observability test results of case II (nonperturbation).

Epoch $t_{k}$	Rank(N)	Observability	1/cond(N)	ln[1/cond(N)]
0	3	False	-	-
1	5	False	-	-
2	7	False	-	-
3	9	False	-	-
⋯	⋯	⋯	⋯	⋯
92	9	False	-	-
93	10	True	$5.1763\times 10^{-14}$	−30.5921
94	10	True	$5.3998\times 10^{-14}$	−30.5498

**Table 4:** Observability test results of case III (with $J_{2}$, $J_{3}$, and $J_{4}$ perturbations).

Epoch $t_{k}$	Rank(N)	Observability	1/cond(N)	ln[1/cond(N)]
0	3	False	-	-
1	5	False	-	-
2	7	False	-	-
3	9	False	-	-
⋯	⋯	⋯	⋯	⋯
488	15	False	-	-
489	16	True	$2.3821\times 10^{-13}$	−29.0656
490	16	True	$2.4174\times 10^{-13}$	−29.0509

**Table 5:** Observability test results of case IV (nonperturbation).

Epoch $t_{k}$	Rank(N)	Observability	1/cond(N)	ln[1/cond(N)]
0	3	False	-	-
1	6	False	-	-
2	8	False	-	-
3	10	False	-	-
⋯	⋯	⋯	⋯	⋯
342	15	False	-	-
343	16	True	$1.9378\times 10^{-13}$	−29.2720
344	16	True	$1.9865\times 10^{-13}$	−29.2472
⋯	⋯	⋯	⋯	⋯
489	16	True	$3.4291\times 10^{-12}$	−26.3987
490	16	True	$3.4823\times 10^{-12}$	−26.3833

**Table 6:** Observability test results of case V.

Epoch $t_{k}$	Rank(N)	Observability	1/cond(N)	ln[1/cond(N)]
0	3	False	-	-
1	6	False	-	-
2	9	False	-	-
3	11	False	-	-
⋯	⋯	⋯	⋯	⋯
1027	18	False	-	-
1028	19	True	$5.7892\times 10^{-13}$	−28.1776
1029	19	True	$5.8186\times 10^{-13}$	−28.1725

**Table 7:** Observability under different situations.

Case	I	II		III		IV	V
Perturbation	*none*	*none*	*none*	$J_{2}$	$J_{2}+J_{3}+J_{4}$	*none*	*none*
Dynamics	Equation (3)	Equation (3)	Equation (3)	Equation (4)	Equation (4)	Equation (3)	Equation (3)
Quaternion of $S_{1}$	**✗**	**✗**	**✗**	**✗**	**✗**	**✗**	**✗**
Orbit state of $S_{1}$	**✓**	**✓**	**✗**	**✗**	**✗**	**✗**	**✗**
Orbit state of $S_{2}$	**✓**	**✗**	**✗**	**✗**	**✗**	**✗**	**✗**
Angular velocity of $S_{1}$	*none*	*none*	*none*	*none*	*none*	**✓**	**✗**
Observability	Y	Y	N	N	Y	Y	Y

## Data Availability

Not applicable.
